# Ruled out of preeclampsia‐like syndrome due to COVID‐19: A case study

**DOI:** 10.1002/ccr3.5195

**Published:** 2021-12-07

**Authors:** Masoumeh Farahani, Kiarash Azadi, Maryam Hashemnejad, Arash Agoushi, Matineh Nirouei

**Affiliations:** ^1^ Department of Obstetrics and Gynecologists Alborz University of Medical Sciences Karaj Iran; ^2^ Alborz University of Medical Sciences Karaj Iran; ^3^ Department of Obstetrics and Gynecologists Alborz University of Medical Sciences Karaj Iran

**Keywords:** case report, delivery, HELLP syndrome, pregnancy, SARS‐CoV‐2, seizure

## Abstract

Coronavirus disease (COVID‐19) is an infectious disease. In this study, we report a 28‐year‐old pregnant woman who had a postpartum seizure with a background of HELLP syndrome and a proven COVID‐19 infection. Her child survived, and at 12‐week postpartum, all maternal COVID‐19–related symptoms vanished, and she was cured.

## Graphical abstract

The study discusses a pregnant woman with postpartum seizure with HELLP syndrome and a COVID‐19 infection. We suspect that COVID‐19 infection in this patient mimics preeclampsia, which progressed to HELLP syndrome and was the possible cause of seizures.

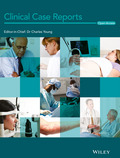

## 1 INTRODUCTION

Coronavirus disease 2019 (COVID‐19) is an infectious disease, which is caused by a new member of a family Coronaviridae named severe acute respiratory syndrome coronavirus 2 (SARS‐CoV‐2), and its primary case series of pneumonia was reported for the first time from Wuhan, Hubei, China, in December 2019.^1^ Hence, pregnant women are more susceptible to viral infections due to immunological alteration during pregnancy, though there is a concern about more susceptibility to SARS‐CoV‐2 infection among pregnant patients. However, based on current evidence, it seems to be more severe in pregnant women in terms of clinical characteristics and susceptibility to SARS‐CoV‐2.^2^, ^3^

Angiotensin‐converting enzyme 2 (ACE2) receptor, which is expressed in vast extra‐pulmonary tissues such as placenta^4^ and brain vascular endothelium,^5^ with the serine protease TMPRSS2, is known as an entrance way for SARS‐CoV‐2 into the cells.^6^ Therefore, it may explain why there are extra‐pulmonary manifestations such as preeclampsia‐like syndrome^7^ or even seizures^8^in patients with COVID‐19.

The aim of this study was to highlight the difficulties in establishing the cause of the postpartum seizure in a case of HELLP (hemolysis, elevated liver enzymes, low platelet) syndrome in patients with a proven COVID‐19 infection.

## 2 PATIENT INFORMATION

The patient was a 28‐year‐old woman, Gravida 2 Parity 1 Live Birth 1, who presented at 38 weeks of gestation with epigastric pain radiating to her back with a history of recent travel and symptoms of subjective fevers with a new sense of smell and taste disorders for 1 week. The patient had received regular prenatal care, and her past obstetric history was uncomplicated, with one full‐term vaginal delivery. She was overweight (body mass index = 25.7) and had no significant past medical history and drug history.

## 3 CLINICAL FINDINGS

Her vital signs were temperature 37.8°C, blood pressure 170/105 mm Hg, heart rate 98 beats per minute, respiratory rate 20 per minute, and oxygen saturation (SpO2) 93% on room air. Fetal status was reassuring by a reactive non‐stress test (NST). In physical examination, respiratory sound was clear bilaterally with soft and non‐tender abdomen.

On the first day of admission, laboratory data showed an elevated white blood cell count and heavy proteinuria; however, other laboratory test results were normal. Other laboratory data are included in Table 1.

**TABLE 1:** Maternal Clinical Laboratory Results

Measure	Reference range	1.5 months earlier	Day 1	Day 2, PPD1	Day 3, PPD2	Day 4, PPD3	Day 5, PPD4	Day 6, PPD5	Day 7, PPD6	Day 8, PPD7
Hemoglobin (g/dl)	12.0–15.5	13.2	13.2	7.22	7.5	9.1	9.0	10.0	9.8	9.6
White blood cell count (×10^9^/L)	3.4–10.0	13.2	18.81	15.61	14.3	18.1	13.2	16.8	15.0	14.2
Lymphocyte count (×10^9^/L)	1.0–3.4	3.03	2.82	2.18	1.43	2.17	NA	1.45	NA	NA
Platelet count (×10^9^/L)	140–450	228	169	41.2	85	96	269	180	305	347
C‐reactive protein (mg/L)	Less than 7.5	NA	NA	151.8	NA	140.9	NA	173.6	NA	300.5
Troponin (μg/L)	Less than 0.05	NA	0.01	Less than 0.02	NA	Less than 0.02	NA	Less than 0.02	Less than 0.02	Less than 0.02
Creatinine (mg/dl)	0.55–1.02	0.8	0.6	0.7	0.8	0.5	0.53	0.57	0.78	0.50
Aspartate transaminase (units/L)	5–44	25	40	72	66	34	27	35	13	13
Alanine transaminase (units/L)	10–61	18	34	67	67	42	24	44	14	14
Alkaline phosphatase (units/L)	38–108	NA	146	113	126	126	69	136	76	74
Prothrombin time (s)	11.7–15.1	NA	11.3	12.4	12.6	12.1	13.4	12.2	14.9	15.6
Ferritin (μg/L)	12–160	NA	NA	187	NA	216	NA	178	NA	224
Lactate dehydrogenase (units/L)	102–199	NA	444.1	846.9	NA	553.6	NA	687.3	NA	319
Fibrin D‐dimers (ng/ml)	Less than 500	NA	NA	1908	808	NA	881	NA	NA	1788
Respiratory viral panel*	NA	NA	NA	NA	NA	NA	NA	NA	NA	NA
25‐hydroxy vitamin D (ng/ml)	20–100	37	NA	NA	NA	NA	NA	NA	NA	NA

Abbreviations: NA, not applicable; PPD, postpartum day.

* Included testing for influenza A and B, adenovirus, metapneumovirus, rhinovirus, and parainfluenza 1–4.

## 4 DIAGNOSIS ASSESSMENT

The patient was considered as a case of severe preeclampsia with suspicion of HELLP syndrome. On second day of her admission, laboratory data results were revealed elevated liver enzymes, serum creatinine, lactate dehydrogenase (LDH), and total and direct bilirubin, and decreased serum albumin, hemoglobin, and platelet count with hyponatremia. Peripheral blood smear examination revealed 1% schistocyte, which lead to administration of two sessions of plasmapheresis on second and third day of her admission.

## 5 THERAPEUTIC INTERVENTION

The patient was considered as a case of severe preeclampsia and started intravenous labetalol (20 mg) and magnesium sulfate (4 g loading dose in 15 min continued by 2 g/h until 8 h postpartum) based on local protocol. On the first day of her admission, the patient was transferred to labor and delivery unit. Hereafter, the cervix was ripped and labor induction was performed by artificial rupture of membranes and administration of oxytocin, which resulted in vaginal delivery of a daughter with an Apgar score of 9/10 at 5 and 10 min. There were no complications during third stage of labor. Thereafter, the patient was transferred to intensive care unit, while her epigastric pain remained with nausea, and a new headache was added to her symptoms. Thus, the internist consult was done during 2 h postpartum; on examination, the patient blood pressure was 157/112 mm Hg along other normal findings. Losartan (50 mg, twice a day), pantoprazole (40 mg, twice a day), and intravenous ondansetron (4 mg) were administered. A request for portable chest radiography for the patient is made, which revealed per bronchial cuffing with increased per hilar markings and raised suspicion of COVID‐19 infection; therefore, nasopharyngeal swab test was obtained and treatment with lopinavir/ritonavir, clindamycin, and dexamethasone was initiated. However, her blood pressure was increased and reached to 185/118 mm Hg; therefore, administration of antihypertensive drugs (such as TNG drip (10 μg/min), hydralazine (5 mg), and labetalol (40 mg) with amlodipine [5 mg, twice a day]) was started. Six hours later, in the morning of her second day of admission, her first seizure occurred despite taking magnesium sulfate and controlled blood pressure. The seizure was a generalized tonic‐colonic seizure with opisthotonic posturing, bleeding due to tongue bite, and drooling. Immediately, midazolam (5 mg) and sodium thiopental (20 mg) were administrated. Then, the airway was secured by intubation because of status seizure. Meanwhile, phenytoin was started for controlling seizure (500 mg loading dose continued by 250 mg, three times a day) and blood samples were sent for laboratory investigation. Following 3 h after first seizure, her second tonic‐colonic seizure occurred. The seizure was controlled by administration of phenytoin (infusion of 1 g in 20 min), and following neurology consultation, magnesium sulfate was continued (1 g/h) for 48 h after seizure with levetiracetam (1.5 g, then 500 mg, twice a day). In neurologic examination, the patient had no response to verbal or painful stimulus, mid‐sized pupils with null reaction to light, and no evidence of papilledema on funduscopy. Liver enzyme rising and schistocyte in peripheral blood smear lead to administration of two sessions of plasmapheresis on the second and third day of her admission. During the third day of admission, the patient was hemodynamically stable despite having bilateral crackles on chest examination. The TTE (trans‐thoracic echocardiography) showed left ventricular ejection fraction of 55% along other normal indices, and concurrent abdominal‐pelvic sonography also showed a mild hydronephrosis in right kidney with mildly enlarged spleen of 62*126 mm without any occupying lesion. The platelet count rose subsequently after plasmapheresis.

Eventually in the fourth day, the respiratory function was restored and the patient extubated. Simultaneously, the positive PCR test result for COVID‐19 was received and the patient was transferred to COVID‐19 referral hospital for continuing treatment.

The patient spent another 2 days in ICU (intensive care unit). Phenytoin, clindamycin, cefotaxime, dexamethasone, and lopinavir/ritonavir were discontinued, and treatment with atazanavir, interferon beta‐1a, and tazocin was started. Also, her antihypertensive drugs were reduced to amlodipine and losartan. Her vital signs on the sixth day of hospitalization were as follows: temperature 37.4°C, blood pressure 117/75 mm Hg, heart rate 94 beats per minute, respiratory rate 18 per minute, and SpO2 98% via face mask. On neurologic examination, the patient just had bilateral 4^+^/5 on lower extremity muscle strength and the rest of examination was normal. Therefore, she transferred to COVID‐19 general ward.

The patient spent seventh and eighth days of her hospitalization in COVID‐19 general ward and underwent a brain MRI (magnetic resonance imaging) for further evaluation, which had no abnormal findings. The patient's laboratory test results were returned to normal with a satisfying medical condition, which led to patient discharge at the end of the eighth day of her hospitalization.

## 6 FOLLOW‐UP AND OUTCOMES

On the follow‐up, at 3 and 12 weeks postpartum, all maternal COVID‐19–related symptoms vanished, and her hypertensive therapy was no longer required. After 12 weeks of delivery, she was not hypertensive.

## 7 DISCUSSION

We present a case of COVID‐19 infection in which the diagnosis of severe preeclampsia was made at presentation but later progressed rapidly into HELLP syndrome and became a magnesium sulfate‐resistant eclampsia at postpartum period coinciding with acute respiratory distress. The patient had two episodes of tonic‐colonic seizure despite having sufficient serum magnesium concentration, and also, her clinical course did not change by delivery. Therefore, this case raises the question of the differential diagnosis between hypertensive disorders and the severe form of COVID‐19 in pregnant women.

In recent publications, findings show that COVID‐19 infection can have several extra‐pulmonary manifestations^6^ such as nausea and/or vomiting, abdominal pain, elevated liver enzymes, thrombocytopenia, and even thrombotic microangiopathy (TMA) as its hematological manifestations that also could be associated with neurologic dysfunction including seizure.^9^, ^10^ Mendoza et al.^7^ proposed that COVID‐19 infection can develop a preeclampsia‐like syndrome that might be distinguished from actual preeclampsia by using angiogenic biomarkers such as sFlt‐1/PlGF along with uterine artery pulsatility index assessment. Furthermore, Sohal et al.^11^ and Karimi et al.^12^ report two cases of COVID‐19 infection with multiple episodes of tonic‐colonic seizures like our patient, but the current literature is too limited. Regarding these considerations, we assumed that our patient's presentation could be a result of cytokine storm due to COVID‐19 infection along with the possibility of encephalitis due to normal brain MRI findings that rolled out the diagnosis of posterior reversible encephalopathy syndrome (PRES) in this patient. Unfortunately, our patient did not undergo CSF fluid analysis, hence, we cannot fully assure about seizures' etiology.

All of the patients with eclampsia have PRESS view in MRI. Besides the manifestations of COVID‐19 infection in this patient, there is one teaching point for clinicians; that is, magnesium sulfate infusion in case of severe PE with COVID‐19 may not have prophylactic effect.

In conclusion, this report indicated that COVID‐19 infection can resemble pregnancy‐related hypertensive disorders with possible CNS involvement, which highlights the need for a cautious approach to pregnancies with suspected preeclampsia and SARS‐CoV‐2 diagnostic testing. Moreover, in order to find the neuroinvasive behaviors of SARS‐CoV‐2 along with neurologic manifestations of COVID‐19 infection, further experimental and clinical studies are needed to be performed.

## 8 PATIENT PERSPECTIVE

The prognosis of postpartum seizure with HELLP syndrome and a COVID‐19 infection in pregnancy was discussed with the patient and her husband. It was explained how complicated this situation was. The patient returned to normal life, and she was very satisfied with the outcome of her treatment.

## CONFLICT OF INTEREST

The authors declare that they have no conflict of interest.

## AUTHOR CONTRIBUTIONS

Study concept and design, Acquisition of data, Drafting of the manuscript, Critical revision of the manuscript for important intellectual content and Administrative, technical, and material support: Masoumeh Farahani, Kiarash Azadi, Maryam Hashemnejad, Arash Agoushi, Matineh Nirouei. Study supervision: Masoumeh Farahani, Maryam Hashemnejad.

## CONSENT

The written informed consent was obtained from the patient.

## Data Availability

The data that support the findings of this study are openly available in public at http://doi.org/10.1002/ccr3.5195, reference number [reference number].
